# Supplementary material for: HMGXB4 Targets Sleeping Beauty Transposition to Germinal Stem Cells
Source: Int J Mol Sci. 2023 Apr 14;24(8):7283. doi: 10.3390/ijms24087283 (PMC10138897; doi:10.3390/ijms24087283)
Supplement: Supplementary file 1 [file ijms-24-07283-s001.zip › Supplementary_Material_HMGXB4.pdf]

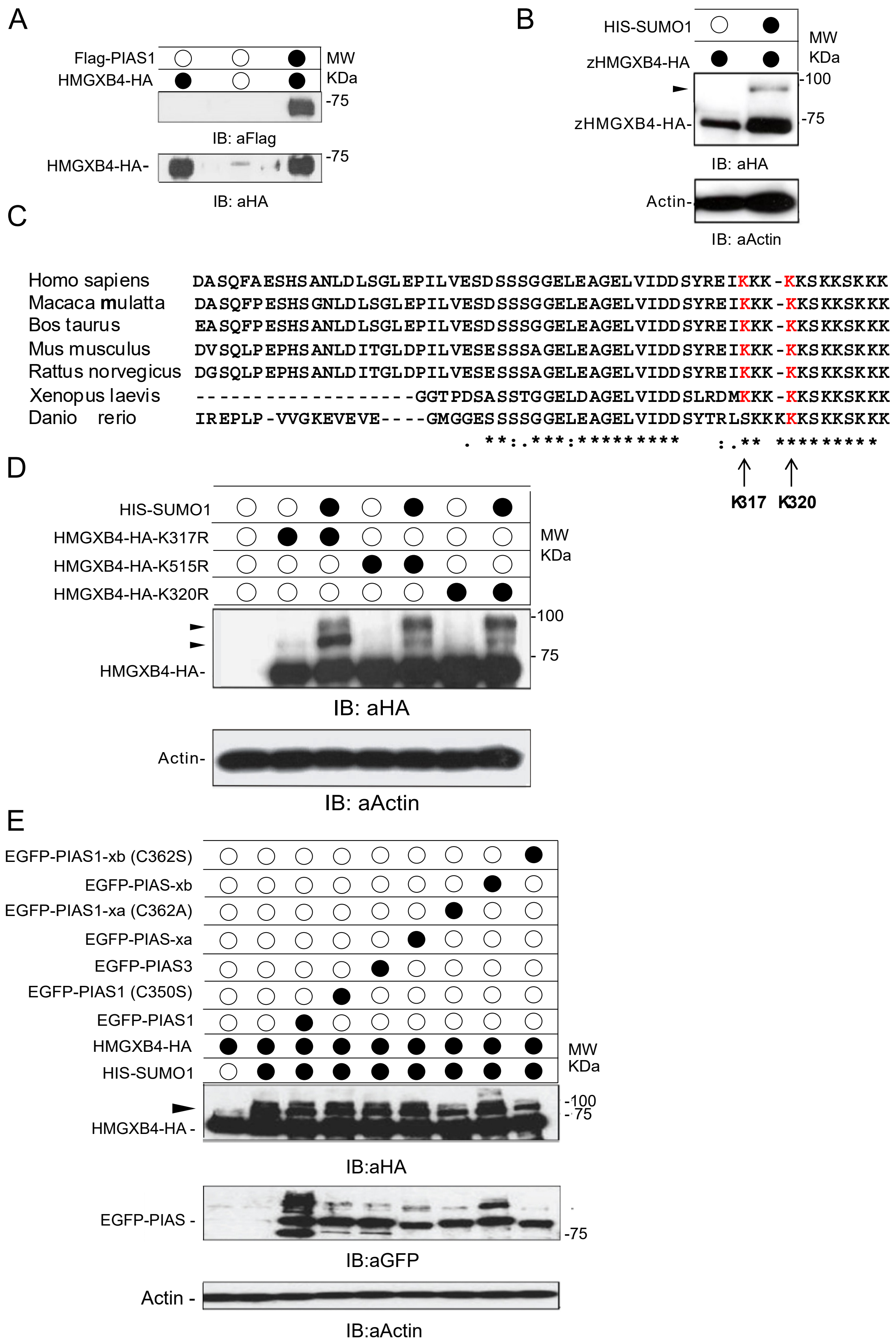

Figure S1. HMGXB4 is Regulated by Post-translational Modification, SUMOylation.

(A) Physical interaction between HMGXB4 and PIAS1. Co-immunoprecipitation of PIAS1 and HMGXB4 in HeLa cell lysates (HMGXB4-HA, PIAS1-FLAG).

(B) HMGXB4 of zebrafish origin (z) gets SUMOylated in the presence of SUMO1 (immunoblots). HeLa cells were co-transfected with expression constructs of the tagged versions of the candidate proteins, zHMGXB4-HA and HIS-SUMO1. Whole-cell lysates were immunoblotted. A slower migrating band, potentially corresponding to the SUMOylated version of HMGXB4, is marked by a black triangle.

(C) Sequence alignment to predict phylogenetically conserved Lysin (K) residues as potential SUMOylation sites in the HMGXB4. The position of the mutated K residues that abolish SUMOylation in HMGXB4 is marked in red. In further studies, the double mutant K317R/K320R is referred to as $HMGXB4^{SUMO}$.

(D) Functional testing of putative SUMOylation mutants. Various mutant versions of HMGXB4 were co-transfected with HIS-SUMO1 into HeLa cells and subjected to immunoblotting using a HA tag-specific antibody. A slower migrating band, potentially corresponding to the SUMOylated version of HMGXB4, is marked by a black triangle.

(E) PIAS does not affect the SUMOylation of HMGXB4. HeLa cells were co-transfected with expression constructs of HMGXB4-HA, HIS-SUMO1, EGFP-PIAS1, PIAS-xa, PIAS-xa and their respective catalytic mutants in various combinations. Whole-cell lysates were subjected to immunoblotting with an anti-HA antibody to detect SUMO modifications, while GFP–specific antibodies were to detect the PIAS proteins. The SUMOylated HMGXB4 is an additional and slower migrating band (marked by a black triangle).

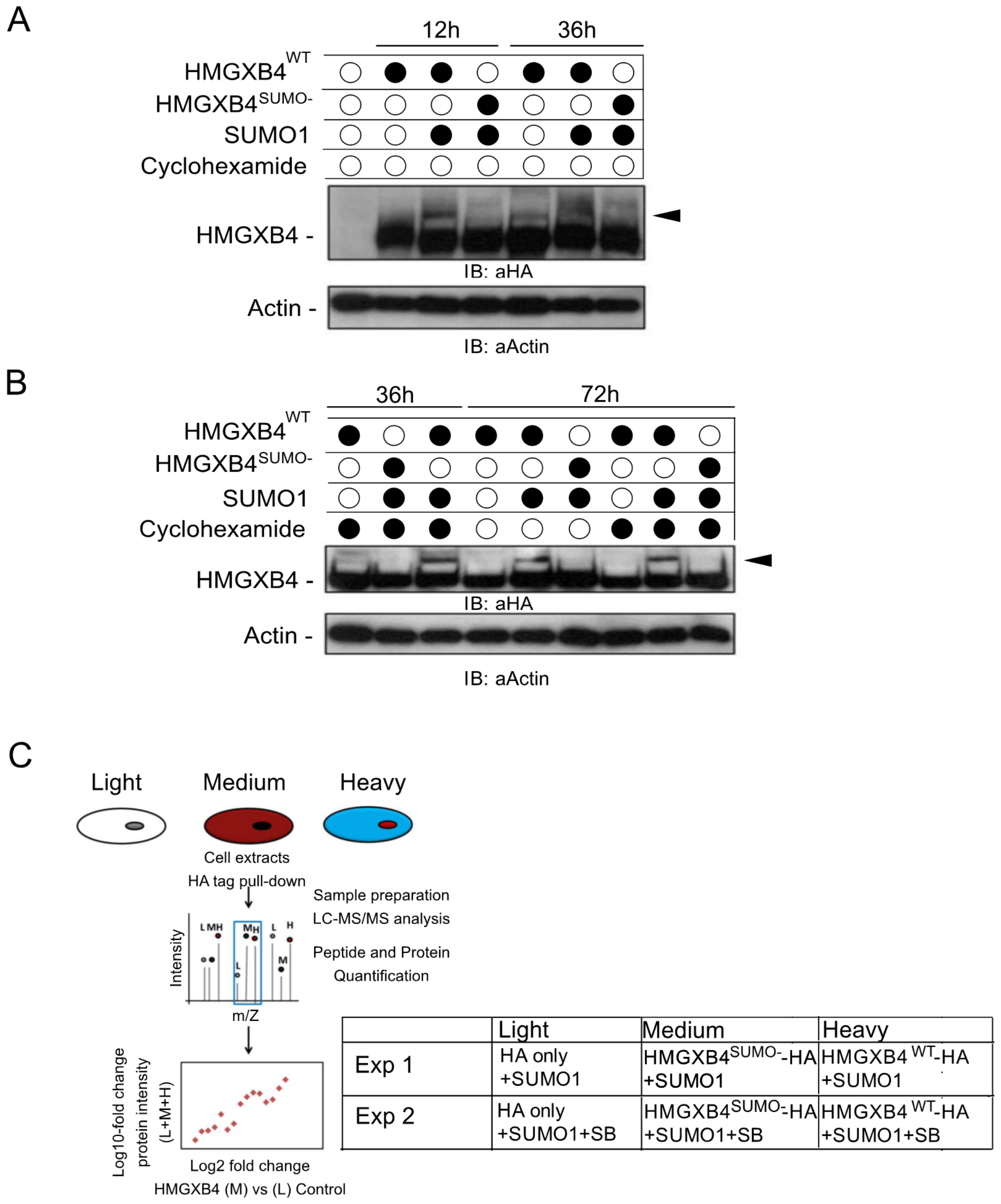

	Light	Medium	Heavy
Exp 1	HA only +SUMO1	HMGXB4^{SUMO-}-HA +SUMO1	HMGXB4^{WT}-HA +SUMO1
Exp 2	HA only +SUMO1+SB	HMGXB4^{SUMO-}-HA +SUMO1+SB	HMGXB4^{WT}-HA +SUMO1+SB

Figure S2. The Effect of SUMOylation on HMGXB4 Function. SUMOylation does not affect the stability of the HMGXB4 protein.

(A-B) SUMOylation does not affect the stability of the HMGXB4 protein. Comparison of steady-state levels of HMGXB4^{WT} and HMGXB4^{SUMO-} (both HA-tagged) in the presence of SUMO1, under conditions, when *de novo* protein synthesis was blocked by cyclohexamide (inhibitor of protein biosynthesis) treatment. HeLa cells were transfected with HA-tagged HMGXB4^{WT} or SUMOylation mutant HMGXB4^{SUMO-} along with SUMO1 in triplicates. Cells were lysed 12, 36 or 72 hours post-transfection without or following cycloheximide treatment. 10 µg of protein were immunoblotted and hybridized with anti-HA antibody. Black triangles mark a slower migrating band, potentially corresponding to the SUMOylated version of HMGXB4.

(C) Triple SILAC pull-down experimental design to investigate the effect of SUMOylation on the interaction partners of HMGXB4 in the absence (Exp 1) or the presence (Exp 2) of the non-hyperactive *Sleeping Beauty* transposase (SB10) [11]. Expression constructs were transiently transfected to HEK293T cells. In the SILAC/pull-down experimental approach, stable isotope labelled amino acids (Light (L) or Medium heavy (M)) is added in the form of a medium supplement to culture HEK293T cells. Detection of interaction partners is performed by mass spectrometry (MS).

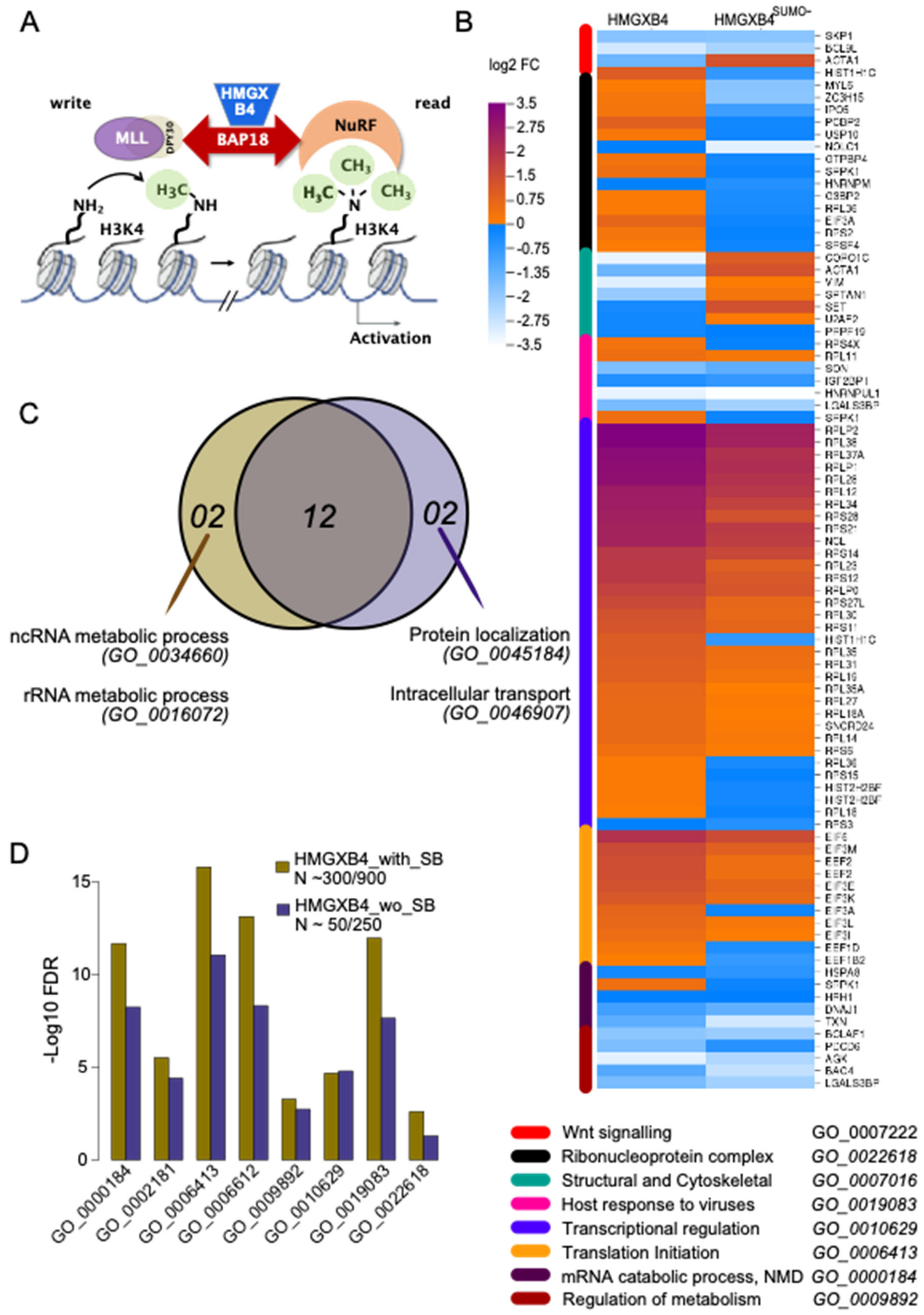

Figure S3. Characterization of the HMGXB4^{WT} and HMGXB4^{SUMO-} Interactomes.

(A) A schematic model shows how C17orf49/BAP18, the interacting partner of HMGXB4, provides a link between NuRF and MLL (via DPY30) complexes (H3K4Me3 mark reader and

writer, respectively [26]) to activate the transcription of a target gene (). H3K4Me3 (histone modification for promoters). The NuRF complex reads the H3K4me3 mark written by the MLL complex and mediates chromatin remodelling.

(B) (Upper panel) Heatmap displaying the similar and distinct patterns of $HMGXB4^{WT}$ and $HMGXB4^{SUMO-}$ interactomes (SILAC-MS). For the differential interaction of C17orf49/BAP18, see Figures 2A and 2C. (Lower panel) Characterization of HMGXB4 interacting protein partners shown on the Heatmap by Gene Ontology (GO) terms. NMD, nonsense-mediated decay.

(C) Venn diagram shows the shared and unique Gene Ontology (GO) terms of the HMGXB4 interacting partners in the presence and absence of SB.

(D) Bar plot showing the shared GO terms HMGXB4 interacting partners in the presence or absence of SB at the level of – Log10 adjusted-p-value (the higher the bar, the lower the false discovery rate). The number of the analysed proteins (N) is indicated next to the colour legends, and the total proteins detected in SILAC experiments were kept as a background. Note the more intense interactome in the presence of the SB transposase in all GO categories.

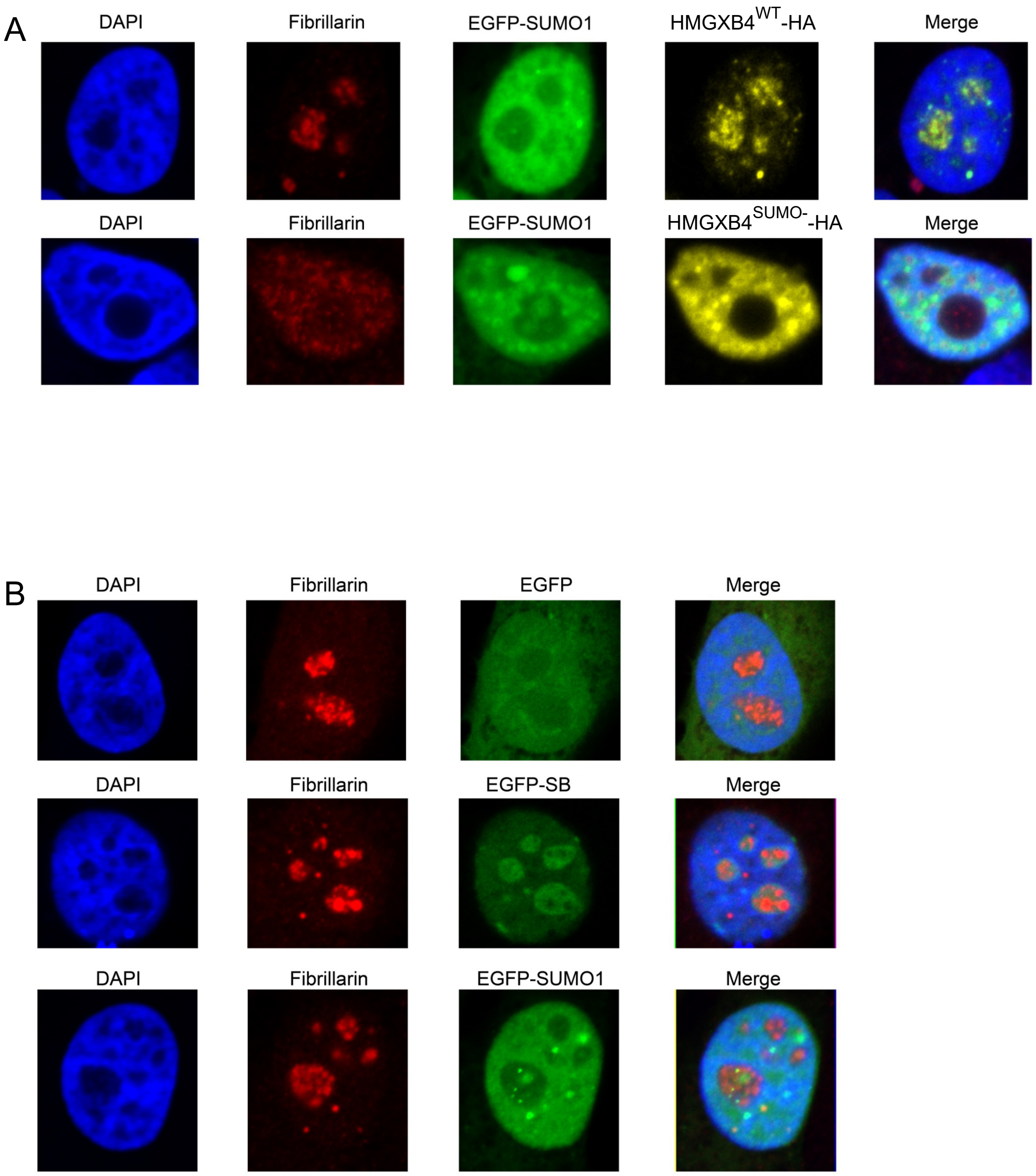

Figure S4. SUMOylated HMGXB4 is compartmentalised to the nucleolus.

(A) Visualization of the sub-nuclear localization of transiently co-expressed EGFP-SUMO1 and (HA-tagged) HMGXB4^{WT} or HMGXB4^{SUMO-}using immuno-fluorescent confocal

microscopy in HeLa cells. EGFP-SUMO1 (green), HMGXB4^{WT}-HA (HA-yellow); DAPI (blue), fibrillarin (red); merge. Note the inverse localization of HMGXB4^{WT} and HMGXB4^{SUMO-}. The similar pattern of HMGXB4^{WT} or HMGXB4^{SUMO-} in the presence or absence of co-transfected SUMO1 suggests that excess of SUMO1 did not change the trafficking behaviour of either version of HMGXB4, meaning that the endogenous SUMO1 is sufficiently marking HMGXB4^{WT} for the nucleolar transit. The expression of HMGXB4^{SUMO-} disrupts the integrity of the nucleolus, which mobilizes the fibrillarin marker all over the cytoplasm (Scale bars 40 μM).

(B) Cellular localisation of EGFP, EGFP-SB and EGFP-SUMO1. Note the nucleolar accumulation of SB transposase (Scale bars 40 μM).

A

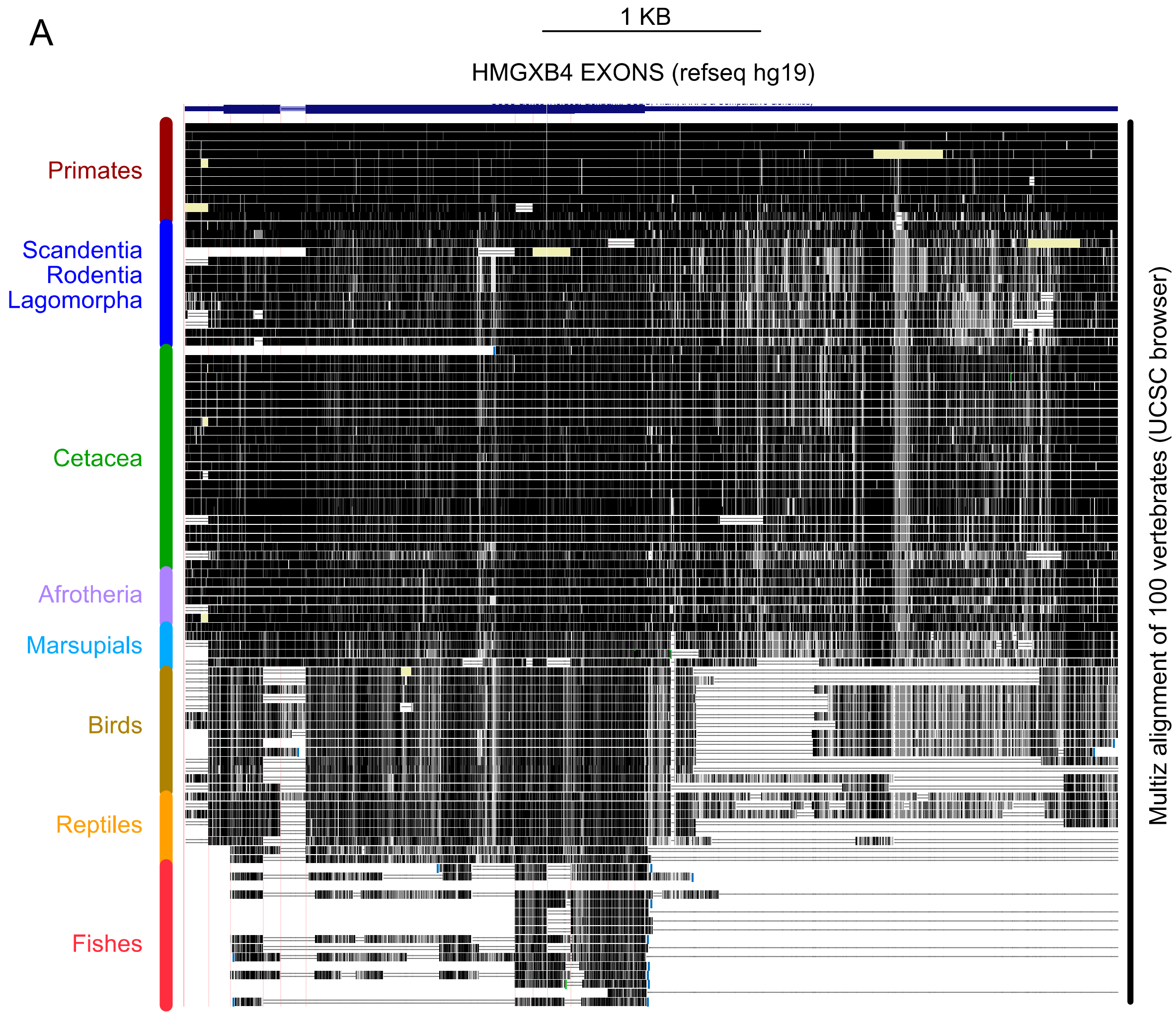

B

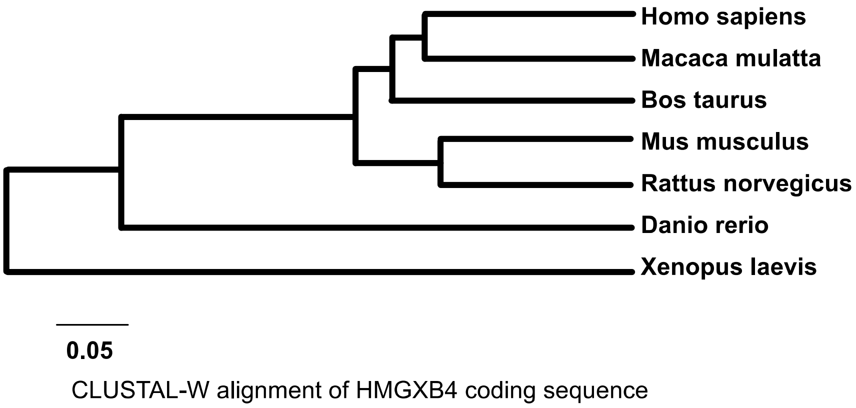

Figure S5. Phylogenetic conservation of HMGXB4 in vertebrates.

(A) Conversation of the HMGXB4 sequences in vertebrates. UCSC snapshot displaying the Multiz alignment of HMGXB4 UTRs and coding sequences across the 100 vertebrates at

single base pair resolution. The black colour denotes the sequence similarity. Gapped sequences are possible deletions when compared to the human version of HMGXB4.

(B) Phylogenetic analysis of HMGXB4 proteins across different vertebrate species. Sequences from various vertebrate species were obtained from NCBI. The sequences were aligned with ClustalW, and the tree was generated using Maximum Parsimony.

Table S1. Predicted SUMOylation sites of the HMGXB4.
In silico predicted SUMOylation sites using (Sumoplot™) www.abgent.com/tools/toSumoplot). The underlined lysine (K) residues were subjected to site-directed mutagenesis to arginine (R) and tested in the *in vitro* SUMOylation assay (co-transfecting them with SUMO1 into HeLa cells and subjected to immunoblotting) (Figure S3D).

Number	Position	Sequence
1	K8	AYDDS VKKE DCFDG
2	K9	YDDSV KKED CFDGD
3	K129	GSKPS KKTG EKSSG
4	K328	SKKSK KKKD KEKHK
5	K392	HSEKK KKKE EKDKE
6	K393	SEKKK KKEE KDKER
7	K501	VLSPQ KKSP PTTML
8	K515	LPASP AKAP ETEPI
